# Multiomics analysis of homologous recombination deficiency across cancer types

**DOI:** 10.17305/bb.2024.10448

**Published:** 2024-07-28

**Authors:** Lin Dong, Lin Li, Linyan Zhu, Fei Xu, Rumeng Zhang, Qiushuang Li, Yong Zhu, Zhutian Zeng, Keshuo Ding

**Affiliations:** 1 Department of Pathology, School of Basic Medical Sciences, Anhui Medical University, Hefei, Anhui, China; 2 Department of Pathology, Tongling People’s Hospital, Tongling, Anhui, China; 3 Department of Pathology, Anhui Provincial Children’s Hospital, Hefei, Anhui, China; 4 Department of Pathophysiology, School of Basic Medical Sciences, Anhui Medical University, Hefei, Anhui, China; 5 Department of Oncology, The First Affiliated Hospital of USTC, Division of Life Sciences and Medicine, University of Science and Technology of China, Hefei, Anhui, China; 6 The CAS Key Laboratory of Innate Immunity and Chronic Disease, School of Basic Medical Sciences, University of Science and Technology of China, Hefei, Anhui, China; 7 Department of Pathology, The First Affiliated Hospital of Anhui Medical University, Hefei, Anhui, China

**Keywords:** Homologous recombination deficiency (HRD), prognosis, gene mutation, DNA methylation, signaling pathway, immunology

## Abstract

There remains ongoing debate regarding the association of homologous recombination deficiency (HRD) with patient survival across various malignancies, highlighting the need for a comprehensive understanding of HRD’s role in different cancer types. Based on data from databases, we conducted a multivariable omics analysis on HRD in 33 cancer types, focusing mainly on 23 cancers in which HRD was significantly associated with patient overall survival (OS) rates. This analysis included the mechanisms related to patient prognosis, gene expression, gene mutation, and signaling pathways. In this study, HRD was found to be significantly associated with patient prognosis, but its impact varied among different cancers. HRD was linked to different outcomes for patients with distinct tumor subtypes and was correlated with clinical features, such as clinical stage and tumor grade. Driver gene mutations, including *TP53*, *MUC4*, *KRAS*, *HRAS*, *FLG*, *ANK3*, *BRCA2*, *ATRX*, *FGFR3*, *NFE2L2*, *MAP3K1*, *PIK3CA*, *CIC*, *FUBP1*, *ALB*, *CTNNB1*, and *MED12*, were associated with HRD across specific cancer types. We also analyzed differentially expressed genes (DEGs) and differentially methylated regions (DMRs) in relation to HRD levels in these cancers. Furthermore, we explored the correlation between HRD and signaling pathways, as well as immune cell infiltration. Overall, our findings contribute to a comprehensive understanding of HRD’s multifaceted role in cancer.

## Introduction

Despite rapid advancements in medical technology, cancer remains a leading global cause of mortality [1]. Recently, immunotherapy has been developed and widely used in cancer treatment, and it is considered one of the most promising strategies for cancer therapy [2–4]. For specific cancer subtypes, such as lung squamous cell carcinoma (LUSC) and bladder urothelial carcinoma (BLCA), immunotherapy has shown significant curative effects and has greatly extended patient survival rates [5, 6]. Despite these significant advances in many cancer subgroups, the clinical application of immunotherapy still faces several challenges related to efficacy and safety [7, 8], and only a small proportion of patients benefit from immunotherapy [9, 10]. For instance, studies have found that anti-programmed cell death protein 1 (anti-PD-1) monoclonal antibodies are a promising treatment for advanced gastric cancer (GC) patients, but the response rate remains limited, and it is necessary to develop new strategies to maximize the efficacy of immune checkpoint inhibitors (ICIs). Therefore, further study to identify precise biomarkers to predict the efficacy of immunotherapy and explore new effective bio-targets for cancer therapy is both important and urgent.

Homologous recombination is a highly conserved process that plays an important role in DNA repair, DNA replication, meiosis, chromosome separation, and telomere maintenance [11]. Homologous recombination repair is one of the core methods for DNA damage repair. It mainly occurs in the S and G2 phases of the cell cycle and serves as a DNA repair mechanism to maintain genome integrity, ensuring the transmission of genetic information with high fidelity [12]. When DNA damage occurs and cannot be repaired normally through the homologous recombination repair process, it is classified as homologous recombination deficiency (HRD) [13]. Some known genes encoding homologous recombination proteins include breast cancer 1 gene (*BRCA1*), breast cancer 2 gene (*BRCA2*), ataxia-telangiectasia mutated gene (*ATM*), ataxia-telangiectasia and Rad3-related gene (*ATR*), BRCA1-associated RING domain 1 gene (*BARD1*), Bloom syndrome protein gene (*BLM*), and the RAD51 recombinase gene (*RAD51*) [14, 15]. As members of homologous recombination proteins, BRCA1 and BRCA2 are widely studied in HRD research due to their correlation with hereditary ovarian and breast cancers [16, 17]. Recent studies have highlighted the relevance of HRD to immunotherapy outcomes and patient prognosis across various cancers [18, 19]. For example, BRCA1/2 mutations play a critical role in stratifying ovarian cancer (OV) subtypes based on HRD scores, influencing treatment decisions and patient outcomes [20]. While extensive research has explored HRD within specific cancers, such as breast, ovarian, and prostate cancers [20–22], comprehensive pan-cancer analyses remain limited. Therefore, there is a compelling need for a detailed investigation into the impact of HRD across diverse cancer types.

Drawing on public databases and bioinformatic methodologies, our study systematically examined the correlation between HRD and patient prognosis, clinical parameters, driver gene mutations, mismatch repair gene (MRG) expression, differentially expressed genes (DEGs), differentially methylated regions (DMRs), signaling pathways, and immune cell infiltration across cancer types. As 23 of the 33 cancer types showed a significant association between HRD and overall survival (OS) rates, this study primarily focused on these 23 cancers (adrenocortical carcinoma [ACC], breast invasive carcinoma [BRCA], colon adenocarcinoma [COAD], esophageal carcinoma [ESCA], head and neck squamous cell carcinoma [HNSC], kidney chromophobe [KICH], kidney renal clear cell carcinoma [KIRC], kidney renal papillary cell carcinoma [KIRP], brain lower grade glioma [LGG], liver hepatocellular carcinoma [LIHC], lung adenocarcinoma [LUAD], mesothelioma [MESO], pancreatic adenocarcinoma [PAAD], pheochromocytoma and paraganglioma [PCPG], rectum adenocarcinoma [READ], sarcoma [SARC], thymoma [THYM], uterine corpus endometrial carcinoma [UCEC], ovarian serous cystadenocarcinoma [OV], BLCA, glioblastoma multiforme [GBM], LUSC, and thyroid carcinoma [THCA]). Our research aims to provide a thorough understanding of HRD’s role and its potential implications for patient prognostication.

## Materials and methods

### Patient cohorts

As in our previous study, we collected patient clinical parameters, HRD levels, tumor subtypes, DNA methylation information, gene mutations, immune cell infiltration data, and other relevant information from public databases, including UCSC-Xena, The Cancer Genome Atlas (TCGA), Firehose case datasets, Tumor Immune Estimation Resource 2.0 (TIMER2.0), and previous publications across 33 cancers [23].

### Calculation of HRD scores

For the entire TCGA cohort, allele-specific copy numbers were estimated from single nucleotide polymorphism (SNP) array data using the Allele-Specific Copy Number Analysis of Tumors (ASCAT) algorithm [24]. The ASCAT estimates were downloaded from the Genomic Data Commons (GDC) Data Portal (https://gdc.cancer.gov) [25]. The HRD scores—telomeric allelic imbalance (TAI) [26], large-scale state transitions (LSTs) [27], loss of heterozygosity (LOH) [28], and the HRDsum [22] (calculated as TAI + LST + LOH)—were computed from allele-specific copy numbers using HRDscar [29]. To compare the HRD scores of TCGA pan-cancer reported in the literature, we downloaded the HRD scores using the UCSCXenaTools R package (https://xenabrowser.net/datapages/), ultimately selecting data from the R package with multiple samples.

### Analysis of the relationship between HRD and patient prognosis /clinical characteristics

For the correlation analysis between HRD and patient prognosis, samples from the 33 cancer types were categorized into high- and low-HRD groups, using the optimal cut-point. Among these 33 cancer types, 23 cancers showed a significant association between HRD and patient OS rates. These 23 cancers included ACC, BLCA, BRCA, COAD, ESCA, GBM, HNSC, KICH, KIRC, KIRP, LGG, LIHC, LUAD, LUSC, MESO, OV, PAAD, PCPG, READ, SARC, THCA, THYM, and UCEC, and they were the focus of subsequent studies. Patient clinical characteristics, including sex, age, race, tumor grade, clinical stage, and smoking status, were included in the correlation study with HRD.

### Gene mutation/MMR expression/DEGs/DNA methylation analyses

Association analyses of gene mutations, MMR expression, DEGs, and DNA methylation with HRD were carried out as described in our previous study [23]. For HRD grouping, the top/bottom one-third method was used. A *P* value of less than 0.05 was considered statistically significant.

### Signaling pathway and immune cell infiltration analysis

Consistent with our previous study, Gene Set Enrichment Analysis (GSEA) 4.0.2 software was used for GSEA, with HRD grouped according to the top/bottom one-third. The “h.all.v7.2.symbols.gmt” gene sets from the Molecular Signatures Database (MSigDB) were used as the reference gene set. A nominal (NOM) *P* value < 0.05 was considered significant for signaling pathway enrichment.

For immune cell infiltration analysis, the Wilcoxon rank-sum test was applied using data from the TCGA database and the ImmuCellAI tool, with HRD grouped by the top/bottom one-third method. Further analysis of immune cell infiltration was conducted using the CIBERSORT algorithm [30].

### Statistical analysis

The correlation between HRD and OS rates was analyzed using the Kaplan–Meier method and Cox regression analysis. PFS rates were also examined using these methods. HRD levels across different cancer subtypes were compared using the Wilcoxon rank-sum test. Additionally, the association between HRD and patient sex was analyzed with the Wilcoxon rank-sum test, while linear regression was used to assess the associations between HRD and other patient characteristics. All statistical analyses were performed using R version 4.0 software (https://www.r-project.org/). A *P* value of less than 0.05 was considered statistically significant.

## Results

### HRD levels and patient prognosis/clinical features association across 33 cancers

Using data from the UCSC-Xena database, we assessed HRD levels across 33 cancer types (ACC, BLCA, BRCA, CESC, CHOL, COAD, DLBC, ESCA, GBM, HNSC, KICH, KIRC, KIRP, LAML, LIHC, LUAD, LGG, LUSC, MESO, OV, PAAD, PCPG, PRAD, READ, SARC, SKCM, STAD, TGCT, UCEC, UCS, UVM, THCA, and THYM). OV exhibited the highest HRD levels, followed by LUSC and ESCA, while THCA showed the lowest HRD levels, followed by renal tumors (LAML, KIRP, KICH, and THYM) (Figure S1).

**Figure 1.**
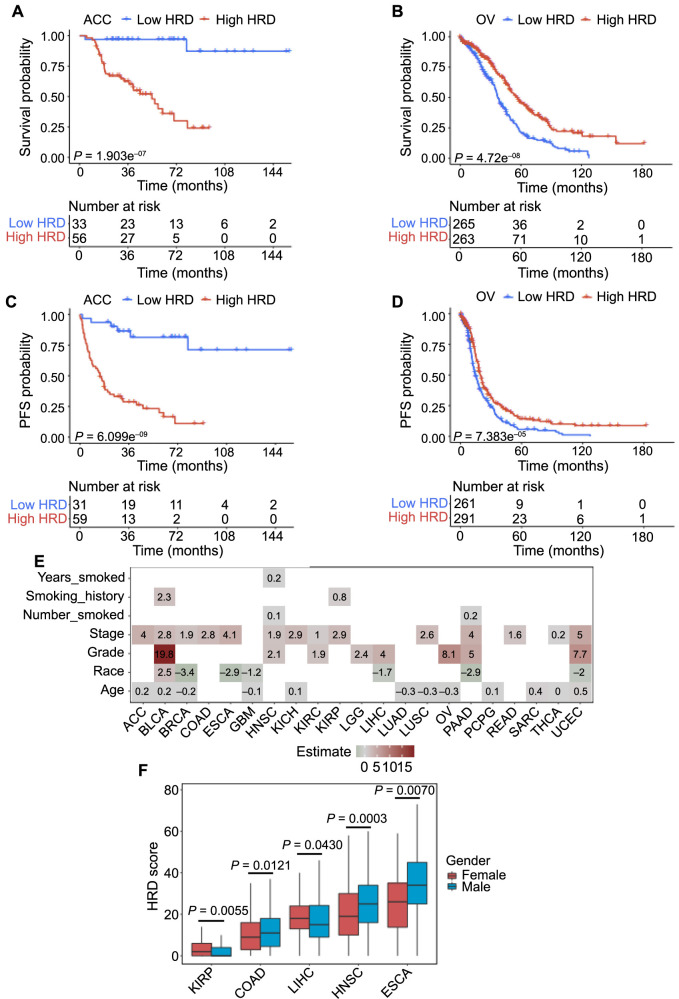
**Association of survival rates and clinical features with HRD in respective cancer types.** (A) Association of OS rate with HRD in patients with ACC; (B) Association of PFS rate with HRD in patients with ACC; (C) Association of OS rate with HRD in patients with OV; (D) Association of PFS rate with HRD in patients with OV; (E) Correlation of patient clinical features including age, race, tumor grade, clinical stage, and smoking status with HRD; (F) HRD levels in male and female patients with different cancers. HRD: Homologous recombination deficiency; OS: Overall survival; ACC: Adrenocortical carcinoma; PFS: Progression-free survival; OV: Ovarian cancer.

We used Kaplan–Meier analysis and the optimal cut-point method to analyze the correlation between HRD and OS rates in the 33 cancer types. As shown in Figure S2, high HRD was associated with lower OS rates in patients with ACC (Figure 1A), BRCA, COAD, ESCA, HNSC, KICH, KIRC, KIRP, LGG, LIHC, LUAD, MESO, PAAD, PCPG, READ, SARC, THYM, and UCEC. In contrast, high HRD was associated with higher OS rates in patients with OV (Figure 1B), BLCA, GBM, LUSC, and THCA. However, the other ten cancer types (LAML, UVM, PRAD, DLBC, TGCT, SKCM, CESC, CHOL, STAD, and UCS) showed no significant correlation between HRD and OS rates. Based on these results, further analysis focused on the 23 cancer types with a significant association between HRD and OS.

Similarly, we performed an analysis to investigate the association between HRD and PFS in the 23 cancers. As shown in Figure S3, high HRD was associated with lower PFS rates in patients with ACC (Figure 1C), BRCA, COAD, ESCA, HNSC, KICH, KIRC, KIRP, LGG, LIHC, MESO, PAAD, PCPG, SARC, THYM, and UCEC, and with higher PFS rates in patients with OV (Figure 1D) and GBM. The associations between HRD and PFS in these 18 cancers were consistent with those observed between HRD and OS. Patients with BLCA, LUAD, LUSC, READ, or THCA showed no significant correlation between HRD and PFS.

In a further analysis, we explored the correlation between cancer subtypes and HRD. As shown in Figure S4, the BRCA basal subtype, GBM IDH-wild type (IDHwt), and UCEC CN-LOW subtype were associated with high HRD levels. The COAD CIN and GS subtypes, ESCA ESCC subtype, HNSC HPV- and HPV+ subtypes, LGG IDHmut-non-codel subtype, READ CIN subtype, and SARC DDLPS and LMS subtypes were associated with low HRD levels.

Additionally, we collected patient clinical information for these 23 cancers and analyzed the correlation with HRD. As shown in Figure 1E, HRD was positively correlated with clinical stages in patients with ACC, BLCA, BRCA, COAD, ESCA, HNSC, KICH, KIRC, KIRP, LUSC, PAAD, READ, THCA, and UCEC. HRD was positively correlated with tumor grades in patients with BLCA, HNSC, KIRC, LGG, LIHC, OV, PAAD, and UCEC, while HRD was negatively correlated with race in patients with BRCA, ESCA, GBM, LIHC, PAAD, and UCEC. Moreover, in several cancer types, HRD levels were associated with gender: male patients with LIHC, COAD, HNSC, and ESCA tended to have high HRD levels, while female patients with KIRP tended to have high HRD levels (Figure 1F).

Therefore, we demonstrated the heterogeneous correlation between patient prognosis/clinical features, and HRD across different cancer types.

### Driver gene mutations and MRG expression associated with HRD

Next, we analyzed the correlation between driver gene mutations/MMR gene expression and HRD in the 23 cancer types.

**Figure 2.**
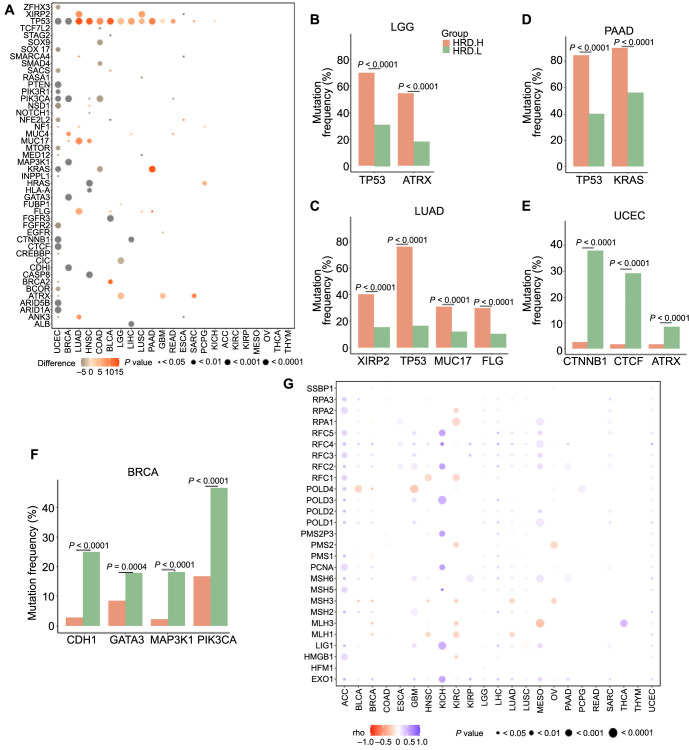
**Association of driver gene mutations and mismatch repair gene expression with HRD.** (A) Association of driver gene mutations with HRD in 23 cancer types, analyzed using the Wilcoxon rank-sum test; (B--F) Driver gene mutations in high-HRD and low-HRD groups for specific cancer types: LGG (B), LUAD (C), PAAD (D), UCEC (E), and BRCA (F); (G) Correlation between the expression of mismatch repair genes and HRD, analyzed by Spearman’s correlation. HRD: Homologous recombination deficiency; LGG: Lower grade glioma; LUAD: Lung adenocarcinoma; PAAD: Pancreatic adenocarcinoma; UCEC: Uterine corpus endometrial carcinoma; BRCA: Breast invasive carcinoma.

As shown in Figure 2A–2D, several driver gene mutations were positively correlated with HRD, including tumor protein 53 (*TP53*) in LUAD (difference ═ 14), HNSC (difference ═ 13), COAD (difference ═ 9), BLCA (difference ═ 13), LGG (difference ═ 4), LIHC (difference ═ 7), LUSC (difference ═ 5), PAAD (difference ═ 10), GBM (difference ═ 1), and READ (difference ═ 7.5); mucin 4 (*MUC4*) in BRCA (difference ═ 8); mucin 17 (*MUC17*) in LUAD (difference ═ 9) and HNSC (difference ═ 8); Kirsten rat sarcoma viral oncogene homolog (*KRAS*) in PAAD (difference ═ 14); Harvey rat sarcoma viral oncogene homolog (*HRAS*) in PCPG (difference ═ 5); filaggrin (*FLG*) in LUAD (difference ═ 6); ankyrin 3 (*ANK3*) in LUAD (difference ═ 8.5); *BRCA2* in BLCA (difference ═ 12.5); and alpha thalassemia/mental retardation syndrome x-linked (*ATRX*) in LGG (difference ═ 4) and SARC (difference ═ 10) (all *P* < 0.01).

However, several driver gene mutations were negatively correlated with HRD, including catenin beta 1 (*CTNNB1*) (difference ═ −18) and CCCTC-binding factor (*CTCF*) (difference ═ −12) in UCEC (Figure 2A and 2E); cadherin 1 (*CDH1*) (difference ═ −13), GATA binding protein 3 (*GATA3*) (difference ═ −6), mitogen-activated protein kinase kinase kinase 1 (*MAP3K1*) (difference ═ −14), and phosphatidylinositol-4,5-bisphosphate 3-kinase catalytic subunit alpha (*PIK3CA*) (difference ═ −12) in BRCA (Figure 2A and 2F); fibroblast growth factor receptor 3 (*FGFR3*) (difference ═ −15) in BLCA; capicua transcriptional repressor (*CIC*) (difference ═ −3) in LGG (Figure 2A); and albumin (*ALB*) (difference ═ −6.5) and catenin beta 1 (*CTNNB1*) (difference ═ −6) in LIHC (Figure 2A) (all *P* < 0.01). In addition, samples of KICH, ACC, KIRC, KIRP, MESO, OV, THCA, and THYM exhibited low HRD levels, with no significant association between HRD and driver gene mutations (Figure 2A).

Loss of function caused by MMR has been proven to induce irreparable DNA replication errors. We used the Spearman method to calculate the correlation between MMR gene expression and HRD. As shown in Figure 2G, in most of the 23 cancers, MMR gene expression was negatively correlated with HRD. Specifically, MMR genes negatively correlated with HRD included the DNA polymerase delta 4, accessory subunit gene (*POLD4*) in BLCA (rho ═ −0.11, *P* value ═ 0.04) and in GBM (rho ═ −0.19, *P* value ═ 0.04); MutL homolog 3 (*MLH3*) in BRCA (rho ═ −0.23, *P* value ═ 1.22E^--12^), in KIRC (rho ═ −0.14, *P* value ═ 0.01), and in MESO (rho ═ −0.23, *P* value ═ 0.05); MutL homolog 3 (*MLH1*) in BRCA (rho ═ −0.18, *P* value ═ 6.39E^--08^), in HNSC (rho ═ −0.12, *P* value ═ 0.01), and in LUAD (rho ═ −0.12, *P* value ═ 0.01). Conversely, we found the MMR gene expression was positively correlated with HRD, including the replication factor C subunit 5 (*RFC5*) in ACC (rho ═ 0.35, *P* value ═ 0.01), in KICH (rho ═ 0.56, *P* value ═ 0.02), and in MESO (rho ═ 0.31, *P* value ═ 0.01); *POLD3* in ACC (rho ═ 0.56, *P* value ═ 2.10E^--05^), in KICH (rho ═ 0.50, *P* value ═ 0.05), and in GBM (rho ═ 0.25, *P* value ═ 0.01); and exonuclease 1 (*EXO1*) in KICH (rho ═ 0.56, *P* value ═ 0.03), in KIRP (rho ═ 0.56, *P* value ═ 1.71E^--07^), and in MESO (rho ═ 0.32, *P* value ═ 0.01) (all *P* < 0.05).

Additionally, we found that *MLH3* was positively correlated with HRD in THCA and UCEC, but negatively correlated with HRD in BRCA, KIRC, and MESO. *POLD4* was positively correlated with HRD in ACC, PCPG, and UCEC, but negatively correlated with HRD in BLCA, BRCA, and HNSC (Figure 2G). However, only 21 out of the 23 cancers showed a significant correlation between MMR gene expression and HRD, with no significant association observed in READ and THYM.

Therefore, these results indicated that the correlation between driver gene mutations and MMR gene expression with HRD varies among different cancer types.

### DEGs and DMRs related to HRD

**Figure 3.**
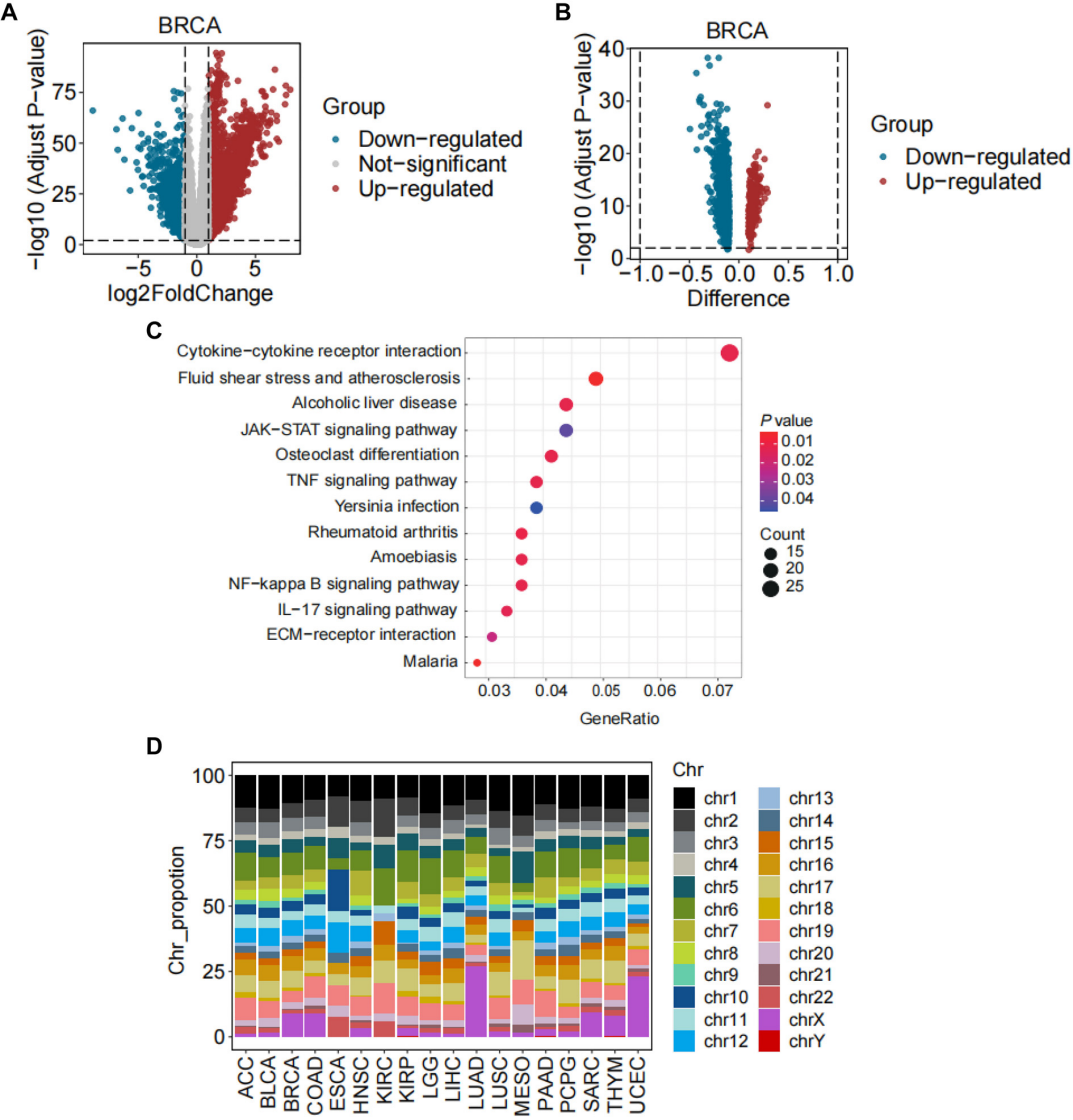
**Association of DEGs and DMRs with HRD.** (A) DEGs in the high-HRD group compared to the low-HRD group in BRCA, with a false discovery rate (FDR) < 0.01 and |log2(FC)| > 1. Upregulated genes are shown in red, non-significant genes in gray, and downregulated genes in blue; (B) DMRs in the promoter regions in the high-HRD group compared to the low-HRD group in BRCA, with FDR < 0.05 and |difference| > 0.1. Upregulated regions are shown in red and downregulated regions in blue; (C) KEGG pathway enrichment associated with DNA methylation in BRCA, illustrated by a bubble plot (all *P* < 0.05); (D) Distribution map of DMRs across respective chromosomes. DEGs: Differentially expressed genes; DMRs: Differentially methylated regions; HRD: Homologous recombination deficiency; BRCA: Breast invasive carcinoma; FDR: False discovery rate; FC: Fold change; KEGG: Kyoto Encyclopedia of Genes and Genomes.

We identified DEGs and DMRs in high- and low-HRD groups (top and bottom one-third by HRD) across 23 cancer types. Among these 23 cancers (Figure 3A, Figure S5), BRCA exhibited the most DEGs and DMRs (Figure 3A and 3B), and the proportion of DEGs related to abnormal methylation was also the highest in BRCA (Table S1). The clustering profile software package of R was used to conduct the Kyoto Encyclopedia of Genes and Genomes (KEGG) analysis of genes related to DNA methylation in BRCA, and a *P* value < 0.05 was considered significant. As shown in Figure 3C and Table S2, 10 of 45 genes were enriched in BRCA, including pathways, such as cytokine–cytokine receptor interaction, fluid shear stress and atherosclerosis, and alcoholic liver disease. For DMR analysis, most DMRs were located on chromosomes 1, 6, and the X chromosome in LUAD and UCEC (Figure 3D and Table S3). However, most DMRs in other cancers were mainly located on chromosomes 1, 2, 6, 8, and 19 (Figure 3D and Table S3). Specifically, in MESO, approximately 15.4% of DMRs were located on chromosome 1; in KIRC, approximately 14.7% of DMRs were located on chromosome 6; and in PAAD, approximately 10.6% of DMRs were located on chromosome 19 (Figure 3D and Table S3). In several cancers, including GBM, KICH, OV, READ, and THCA, no DMRs were found. Overall, while gene expression differences were widely observed between high- and low-HRD groups across different cancers, the frequency of differential methylation in promoter regions was lower.

### Signaling pathway and immune cell infiltration associated with HRD

To better understand the impact of HRD on cell signaling pathways and the tumor microenvironment, we used the top/bottom one-third method for analyses.

**Figure 4.**
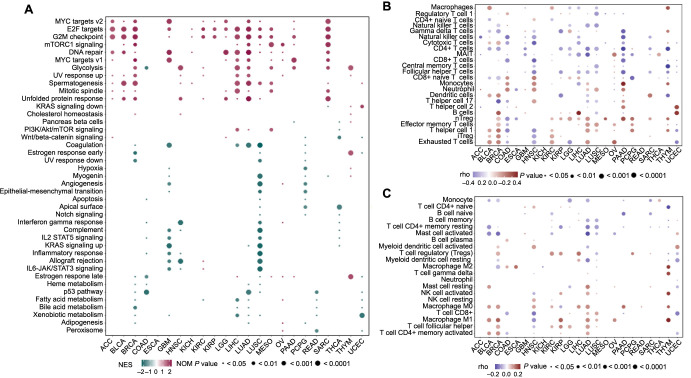
**Association of signaling pathways and immune cell infiltration with HRD.** (A) Signaling pathways associated with HRD in 23 cancer types, analyzed using the Wilcoxon rank-sum test; (B and C) Immune cell infiltration related to HRD in 23 cancer types, analyzed using the Wilcoxon rank-sum test based on the ImmuCellAI algorithm (B) and the CIBERSORT algorithm (C), respectively. HRD: Homologous recombination deficiency; ImmuCellAI: Immune cell abundance identifier; CIBERSORT: Cell-type identification by estimating relative subsets of RNA transcripts.

In the signaling pathway analysis, as shown in Figure 4A, we found that the G2M checkpoint, E2F targets, and DNA repair pathways were enriched in the high-HRD groups of ACC, BLCA, BRCA, GBM, LGG, LIHC, LUAD, LUSC, MESO, and SARC. Conversely, the coagulation and P53 pathways were enriched in the low-HRD groups of BRCA, LUAD, and LUSC. However, in many cancer types, the signaling pathway enrichments in samples with different HRD levels were more complex. For example, the glycolysis pathway was enriched in the high-HRD groups of BRCA, HNSC, KIRC, LIHC, LUAD, MESO, PAAD, SARC, and THYM, but was enriched in low-HRD group of COAD. The estrogen response late pathway was enriched in the high-HRD groups of THYM, PAAD, OV, MESO, and LIHC, but was enriched in the low-HRD groups of BRCA, COAD, LUSC, and UCEC. The allograft rejection pathway was enriched in the high-HRD group of KIRC, but was enriched in low HRD groups of GBM, HNSC, and LUSC. Therefore, HRD-associated cell signaling pathways varied across different cancers, although the G2M checkpoint, E2F targets, and DNA repair pathways appeared to be important in many HRD-associated cancers.

Using the ImmuCellAI tool and the CIBERSORT algorithm, we investigated the relationship between immune cell infiltration and HRD. Heatmaps showed that immune cell infiltration, including macrophages, T follicular helper (Tfh) cells, CD4+ memory-activated T cells, T helper 1 (Th1) cells, regulatory T cells (nTreg, iTreg), and monocytes, was positively associated with HRD. Conversely, immune cell infiltration of CD4+ T cells, naive CD4+ T cells, naive B cells, and activated mast cells was negatively associated with HRD. Additionally, macrophage M0 infiltration was positively associated with HRD in BLCA, BRCA, HNSC, KIRC, LIHC, LUAD, PAAD, and THYM, but negatively associated with HRD in KIRP. Macrophage M1 infiltration was positively associated with HRD in BLCA, BRCA, KIRP, LUAD, LUSC, OV, and THYM, but negatively associated with HRD in HNSC (Figure 4B and 4C, Table S4). Therefore, the correlation between HRD and immune cell infiltration varied among different cancer types.

## Discussion

Historically, studies on HRD have predominantly focused on specific cancer types, such as breast, ovarian, and prostate cancers [20–22, 31]. Comprehensive pan-cancer analyses of HRD across diverse malignancies remain limited. Using data from public databases, we conducted a multivariable omics analysis encompassing 33 cancer types, with a particular focus on 23 cancers where HRD significantly correlated with OS rates. This analysis included mechanisms related to patient prognosis, gene expression, gene mutation, gene methylation, and signaling pathways. Our study uncovered the roles of HRD in different cancer types and highlighted possible reasons for its correlation with patient prognosis.

Through Kaplan–Meier analysis, we found that in most cancers (including ACC, BRCA, COAD, ESCA, HNSC, KICH, KIRC, KIRP, LGG, LIHC, MESO, PAAD, PCPG, SARC, THYM, and UCEC), high levels of HRD were correlated with both lower OS and PSF rates. As previously reported, high-HRD patients with LGG had significantly worse OS compared to low-HRD patients [32]. In BRCA, HRD-high tumors were more clinically aggressive and associated with a higher risk of recurrence, especially in estrogen receptor-positive (ER+) tumors [33]. Additionally, in cancers, such as ACC, STAD, UCEC, KIRC, SARC, PRAD, PAAD, and BRCA, patients with high HRD scores exhibited worse prognoses than those with low HRD scores [34–38], consistent with our data. Conversely, in OV and GBM patients, high levels of HRD were correlated with both higher OS and PFS rates, concordant with the studies by Knijnenburg et al. [39] and Shi et al. [40]. High-HRD tumors have been shown to respond better to platinum-based chemotherapies, particularly in high-grade serous OV [41–43]. Furthermore, we revealed that in cancers, such as BLCA, HNSC, KIRC, PAAD, and UCEC, high levels of HRD were associated with higher clinical stages and tumor grades. As reported in previous studies, in HNSC patients, clinical stage, clinical T stage, pathological T stage, and lymphovascular invasion were associated with a high HRD score (HRD-H), and HRD-H was linked to poor outcomes [44]. Similarly, in kidney renal clear cell carcinoma and endometrial cancer, patients with high HRD exhibited worse prognosis [45, 46]. Therefore, HRD is an important factor in predicting patient prognosis, with correlations that vary among cancer types.

In the analysis of driver gene mutations and MMR gene expression, we found that many driver gene mutations, including *TP53*, were positively correlated with HRD in cancers such as LUAD, HNSC, COAD, BLCA, LGG, LIHC, LUSC, PAAD, GBM, READ, and SARC. Mutations in the tumor suppressor *TP53* gene are among the most common genetic alterations in human cancers [47, 48], and *TP53* alterations have been associated with higher HRD scores [39, 49]. For example, in LIHC, up to 30% of patients carry *TP53* mutations, which act as a significant risk factor [50, 51]. In NSCLC, *TP53* alterations have been correlated with poorer OS rates and greater resistance to chemotherapy and radiation [47, 52]. Furthermore, *TP53* mutations have been linked to poor prognosis in HNSC, COAD, BLCA, LGG, GBM, and SARC [53–57]. Thus, the poor prognosis in high-HRD cancer patients may be attributed to the high frequency of *TP53* mutations. We also demonstrated that *KRAS* mutations were positively correlated with HRD in PAAD. *KRAS* mutations are present in approximately 75% of pancreatic ductal adenocarcinoma cases and are associated with worse outcomes [58, 59]. Additionally, our data showed a positive correlation between *BRCA2* mutations and HRD in BLCA. While *BRCA2* mutations contribute to genomic instability and malignant transformation [60], previous studies have found that the *BRCA2* gene can inhibit the occurrence and development of cancer. Patients with *BRCA2* mutations tend to be more sensitive to chemotherapy and radiotherapy, leading to better prognoses in certain cancers, such as breast, ovarian, and bladder cancers [60–64]. Our analysis found that high HRD was associated with higher OS rates in OV and BLCA patients, aligning with these findings.

In the present study, we observed that immune cell infiltration, including macrophages, Tfh cells, CD4+ memory-activated T cells, T helper cell 1 (Th1), regulatory T cells (nTreg, iTreg), and monocytes, was positively associated with HRD, while CD4+ T cells, naive CD4+ T cells, naive B cells, and activated mast cells were negatively associated with HRD. Previous research has shown that tumors with high HRD scores exhibit increased leukocyte infiltration and an immune-sensitive microenvironment [65]. In BRCA patients, high infiltration of immune cells, including tumor-associated macrophages (TAMs), neutrophils, Tregs, and myeloid-derived suppressor cells (MDSCs), has been correlated to worse cumulative survival rates. Macrophages M2, neutrophils, and Tregs infiltration has been negatively correlated with prognosis in colorectal cancer patients [66]. Tregs are known to suppress antitumor immunity and deter immune surveillance, contributing to poor prognosis in various cancers, including breast and colorectal cancers [67–69]. Increased numbers of Tregs and TAMs have also been associated with poor prognosis in NSCLC, LIHC, and clear cell renal cell carcinoma [70–72]. Therefore, TAMs and Tregs infiltration may mediate the relationship between HRD and poor prognosis in cancers such as BRCA, COAD, KIRC, LUAD, and LIHC. However, in contrast to our HRD-related data, previous studies have shown that Tfh cell infiltration is associated with favorable outcomes in lung adenocarcinoma, breast cancer, and colorectal cancer [73–75], suggesting that Tfh cells may play a minor role in the context of HRD compared to other immune cells.

## Conclusion

In conclusion, our pan-cancer analysis highlights the heterogeneous impact of HRD on patient prognosis, genetic alterations, and the immune microenvironment across 33 cancer types. These findings underscore HRD as a critical biomarker for predicting clinical outcomes and guiding personalized treatment strategies in oncology. Future studies integrating multiomics approaches and prospective clinical trials are warranted to validate our observations and translate them into improved patient care.

## Supplemental data

Supplemental tables are available at the following link:

https://www.bjbms.org/ojs/index.php/bjbms/article/view/10448/3393

Supplemental figures are available at the following link:

https://www.bjbms.org/ojs/index.php/bjbms/article/view/10448/3391

The legends for the supplemental figures are provided in the following text:

**Figure S1.** **HRD levels calculated across the 33 cancer types.** HRD: Homologous recombination deficiency.

**Figure S2.** **Association of OS rates with HRD across various cancer types.** HRD: Homologous recombination deficiency; OS: Overall survival.

**Figure S3.** **Association of PFS rates with HRD across various cancer types.** HRD: Homologous recombination deficiency; PFS: Progression-free survival.

**Figure S4.** **Association of OS rates with HRD in 13 molecular subtypes across nine different cancers.** HRD: Homologous recombination deficiency; OS: Overall survival.

**Figure S5.** **Volcano plot showing DEGs in the high-HRD group compared to the low-HRD group across 22 cancer types.** DEGs were determined with an FDR of < 0.01 and a log2 fold change (|log2(FC)|) > 1. Upregulated genes are shown in red, downregulated in blue, and non-significant genes in gray. HRD: Homologous recombination deficiency; DEGs: Differentially expressed genes; FDR: False discovery rate.

## Data Availability

All relevant data are available within the article and its supplementary data.

## References

[1] Rommasi F (2022). Bacterial-based methods for cancer treatment: what we know and where we are. Oncol Ther.

[2] Brahmer J, Reckamp KL, Baas P, Crinò L, Eberhardt WE, Poddubskaya E (2015). Nivolumab versus docetaxel in advanced squamous-cell non-small-cell lung cancer. N Engl J Med.

[3] Powles T, Eder JP, Fine GD, Braiteh FS, Loriot Y, Cruz C (2014). MPDL3280A (anti-PD-L1) treatment leads to clinical activity in metastatic bladder cancer. Nature.

[4] Robert C, Ribas A, Wolchok JD, Hodi FS, Hamid O, Kefford R (2014). Anti-programmed-death-receptor-1 treatment with pembrolizumab in ipilimumab-refractory advanced melanoma: a randomised dose-comparison cohort of a phase 1 trial. Lancet (London, England).

[5] Johnson DB, Nebhan CA, Moslehi JJ, Balko JM (2022). Immune-checkpoint inhibitors: long-term implications of toxicity. Nat Rev Clin Oncol.

[6] Galluzzi L, Humeau J, Buqué A, Zitvogel L, Kroemer G (2020). Immunostimulation with chemotherapy in the era of immune checkpoint inhibitors. Nat Rev Clin Oncol.

[7] Tang S, Qin C, Hu H, Liu T, He Y, Guo H (2022). Immune checkpoint inhibitors in non-small cell lung cancer: progress, challenges, and prospects. Cells.

[8] Kono K, Nakajima S, Mimura K (2020). Current status of immune checkpoint inhibitors for gastric cancer. Gast Canc.

[9] Chen L, Zhou Q, Liu J, Zhang W (2021). CTNNB1 alternation is a potential biomarker for immunotherapy prognosis in patients with hepatocellular carcinoma. Front Immunol.

[10] Lonser RR, Song DK, Klapper J, Hagan M, Auh S, Kerr PB (2011). Surgical management of melanoma brain metastases in patients treated with immunotherapy. J Neurosurg.

[11] Gonzalez D, Stenzinger A (2021). Homologous recombination repair deficiency (HRD): from biology to clinical exploitation. Genes Chromosomes Cancer.

[12] Stover EH, Fuh K, Konstantinopoulos PA, Matulonis UA, Liu JF (2020). Clinical assays for assessment of homologous recombination DNA repair deficiency. Gynecol Oncol.

[13] Doig KD, Fellowes AP, Fox SB (2023). Homologous recombination repair deficiency: an overview for pathologists. Mod Pathol.

[14] Roy R, Chun J, Powell SN (2011). BRCA1 and BRCA2: different roles in a common pathway of genome protection. Cancer.

[15] Cruz C, Castroviejo-Bermejo M, Gutiérrez-Enríquez S, Llop-Guevara A, Ibrahim YH, Gris-Oliver A (2018). RAD51 foci as a functional biomarker of homologous recombination repair and PARP inhibitor resistance in germline BRCA-mutated breast cancer. Ann Oncol.

[16] Mavaddat N, Peock S, Frost D, Ellis S, Platte R, Fineberg E (2013). Cancer risks for BRCA1 and BRCA2 mutation carriers: results from prospective analysis of EMBRACE. J Natl Cancer Inst.

[17] Venkitaraman AR (2014). Cancer suppression by the chromosome custodians, BRCA1 and BRCA2. Science.

[18] Liu X, Wang T, Ren Z, Feng C, Tian X (2023). Identification of novel prognostic model based on homologous recombination deficiency associated lncRNAs in lung adenocarcinoma. Heliyon.

[19] Wang H, Gong F, Kong W, Chen Y, Zhang J (2023). Homologous recombination repair gene-based risk model predicts prognosis and immune microenvironment for primary lung cancer after previous malignancies. J Gene Med.

[20] Sztupinszki Z, Diossy M, Borcsok J, Prosz A, Cornelius N, Kjeldsen MK (2021). Comparative assessment of diagnostic homologous recombination deficiency-associated mutational signatures in ovarian cancer. Clin Cancer Res.

[21] Zhuang S, Chen T, Li Y, Wang Y, Ai L, Geng Y (2021). A transcriptional signature detects homologous recombination deficiency in pancreatic cancer at the individual level. Mol Ther Nucleic Acids.

[22] Telli ML, Timms KM, Reid J, Hennessy B, Mills GB, Jensen KC (2016). Homologous recombination deficiency (HRD) score predicts response to platinum-containing neoadjuvant chemotherapy in patients with triple-negative breast cancer. Clin Cancer Res.

[23] Li L, Bai L, Lin H, Dong L, Zhang R, Cheng X (2021). Multiomics analysis of tumor mutational burden across cancer types. Comput Struct Biotechnol J.

[24] Van Loo P, Nordgard SH, Lingjærde OC, Russnes HG, Rye IH, Sun W (2010). Allele-specific copy number analysis of tumors. Proc Natl Acad Sci U S A.

[25] Grossman RL, Heath AP, Ferretti V, Varmus HE, Lowy DR, Kibbe WA (2016). Toward a shared vision for cancer genomic data. N Engl J Med.

[26] Birkbak NJ, Wang ZC, Kim JY, Eklund AC, Li Q, Tian R (2012). Telomeric allelic imbalance indicates defective DNA repair and sensitivity to DNA-damaging agents. Cancer Discov.

[27] Popova T, Manié E, Rieunier G, Caux-Moncoutier V, Tirapo C, Dubois T (2012). Ploidy and large-scale genomic instability consistently identify basal-like breast carcinomas with BRCA1/2 inactivation. Cancer Res.

[28] Abkevich V, Timms KM, Hennessy BT, Potter J, Carey MS, Meyer LA (2012). Patterns of genomic loss of heterozygosity predict homologous recombination repair defects in epithelial ovarian cancer. Br J Cancer.

[29] Sztupinszki Z, Diossy M, Krzystanek M, Reiniger L, Csabai I, Favero F (2018). Migrating the SNP array-based homologous recombination deficiency measures to next generation sequencing data of breast cancer. NPJ Breast Cancer.

[30] Miao YR, Zhang Q, Lei Q, Luo M, Xie GY, Wang H (2020). ImmuCellAI: a unique method for comprehensive T-cell subsets abundance prediction and its application in cancer immunotherapy. Adv Sci (Weinh).

[31] Lotan TL, Kaur HB, Salles DC, Murali S, Schaeffer EM, Lanchbury JS (2021). Homologous recombination deficiency (HRD) score in germline BRCA2- versus ATM-altered prostate cancer. Mod Pathol.

[32] Peng H, Wang Y, Wang P, Huang C, Liu Z, Wu C (2022). A risk model developed based on homologous recombination deficiency predicts overall survival in patients with lower grade Glioma. Front Genet.

[33] Walens A, Van Alsten SC, Olsson LT, Smith MA, Lockhart A, Gao X (2022). RNA-Based classification of homologous recombination deficiency in racially diverse patients with breast cancer. Cancer Epidemiol Biomarkers Prev.

[34] Wu X, Wang Q, Liu P, Sun L, Wang Y (2022). Potential value of the homologous recombination deficiency signature we developed in the prognosis and drug sensitivity of gastric cancer. Front Genet.

[35] Liu X, Jiang S, Wang H, Wu X, Yan W, Chen Y (2022). Pegylated liposomal doxorubicin combined with ifosfamide for treating advanced or metastatic soft-tissue sarcoma: a prospective, single-arm phase II study. Clin Cancer Res.

[36] Nacev BA, Sanchez-Vega F, Smith SA, Antonescu CR, Rosenbaum E, Shi H (2022). Clinical sequencing of soft tissue and bone sarcomas delineates diverse genomic landscapes and potential therapeutic targets. Nat Commun.

[37] Pokataev I, Fedyanin M, Polyanskaya E, Popova A, Agafonova J, Menshikova S (2020). Efficacy of platinum-based chemotherapy and prognosis of patients with pancreatic cancer with homologous recombination deficiency: comparative analysis of published clinical studies. ESMO Open.

[38] Wattenberg MM, Reiss KA (2021). Determinants of homologous recombination deficiency in pancreatic cancer. Cancers (Basel).

[39] Knijnenburg TA, Wang L, Zimmermann MT, Chambwe N, Gao GF, Cherniack AD (2018). Genomic and molecular landscape of DNA damage repair deficiency across the cancer genome atlas. Cell Rep.

[40] Shi Z, Chen B, Han X, Gu W, Liang S, Wu L (2023). Genomic and molecular landscape of homologous recombination deficiency across multiple cancer types. Sci Rep.

[41] Przybytkowski E, Davis T, Hosny A, Eismann J, Matulonis UA, Wulf GM (2020). An immune-centric exploration of BRCA1 and BRCA2 germline mutation related breast and ovarian cancers. BMC Cancer.

[42] Patel JN, Braicu I, Timms KM, Solimeno C, Tshiaba P, Reid J (2018). Characterisation of homologous recombination deficiency in paired primary and recurrent high-grade serous ovarian cancer. Br J Cancer.

[43] do Canto LM, Larsen SJ, Catin Kupper BE, Begnami M, Scapulatempo-Neto C, Petersen AH (2019). Increased levels of genomic instability and mutations in homologous recombination genes in locally advanced rectal carcinomas. Front Oncol.

[44] Chen Y, Zheng X, Lin J, Gao X, Xiong J, Liu J (2022). A high homologous recombination deficiency score is associated with poor survival and a non-inflamed tumor microenvironment in head and neck squamous cell carcinoma patients. Oral Oncol.

[45] He L, Gao F, Zhu J, Xu Q, Yu Q, Yang M (2023). Homologous recombination deficiency serves as a prognostic biomarker in clear cell renal cell carcinoma. Exp Ther Med.

[46] Siedel JH, Ring KL, Hu W, Dood RL, Wang Y, Baggerly K (2021). Clinical significance of homologous recombination deficiency score testing in endometrial Cancer. Gynecol Oncol.

[47] Mogi A, Kuwano H (2011). TP53 mutations in nonsmall cell lung cancer. J Biomed Biotechnol.

[48] Olivier M, Hussain SP, Caron de Fromentel C, Hainaut P, Harris CC (2004). TP53 mutation spectra and load: a tool for generating hypotheses on the etiology of cancer. IARC Sci Publ.

[49] Ciriello G, Miller M L, Aksoy B A, Senbabaoglu Y, Schultz N, Sander C (2013). Emerging landscape of oncogenic signatures across human cancers. Nat Genet.

[50] Liu J, Ma Q, Zhang M, Wang X, Zhang D, Li W (2012). Alterations of TP53 are associated with a poor outcome for patients with hepatocellular carcinoma: evidence from a systematic review and meta-analysis. Eur J Cancer.

[51] Luo K, Qian Z, Jiang Y, Lv D, Zhu K, Shao J (2023). Characterization of the metabolic alteration-modulated tumor microenvironment mediated by TP53 mutation and hypoxia. Comput Biol Med.

[52] Peng J, Zhan Y, Feng J, Fan S, Zang H (2019). Expression of WDR79 is associated with TP53 mutation and poor prognosis in surgically resected non-small cell lung cancer. J Cancer.

[53] Li L, Li M, Wang X (2020). Cancer type-dependent correlations between TP53 mutations and antitumor immunity. DNA Repair.

[54] Wu X, Lv D, Cai C, Zhao Z, Wang M, Chen W (2020). A TP53-associated immune prognostic signature for the prediction of overall survival and therapeutic responses in muscle-invasive bladder cancer. Front Immunol.

[55] Park Y, Park J, Ahn JW, Sim JM, Kang SJ, Kim S (2021). Transcriptomic landscape of lower grade Glioma based on age-related non-silent somatic mutations. Curr Oncol.

[56] Ohgaki H, Kleihues P (2013). The definition of primary and secondary glioblastoma. Clin Cancer Res.

[57] Tirode F, Surdez D, Ma X, Parker M, Le Deley MC, Bahrami A (2014). Genomic landscape of Ewing sarcoma defines an aggressive subtype with co-association of STAG2 and TP53 mutations. Cancer Discov.

[58] McIntyre CA, Lawrence SA, Richards AL, Chou JF, Wong W, Capanu M (2020). Alterations in driver genes are predictive of survival in patients with resected pancreatic ductal adenocarcinoma. Cancer.

[59] Kim J, Reber HA, Dry SM, Elashoff D, Chen SL, Umetani N (2006). Unfavourable prognosis associated with K-ras gene mutation in pancreatic cancer surgical margins. Gut.

[60] Kuang S, Li H, Feng J, Xu S, Le Y (2019). Correlation of BRCA2 gene mutation and prognosis as well as variant genes in invasive urothelial carcinoma of the bladder. Cancer Biomark.

[61] Chen PL, Chen CF, Chen Y, Xiao J, Sharp ZD, Lee WH (1998). The BRC repeats in BRCA2 are critical for RAD51 binding and resistance to methyl methanesulfonate treatment. Proc Natl Acad Sci USA.

[62] Shao J, Yang J, Wang JN, Qiao L, Fan W, Gao QL (2015). Effect of BRCA2 mutation on familial breast cancer survival: a systematic review and meta-analysis. J Huazhong Univ Sci Technolog Med Sci.

[63] Parvin S, Islam M S, Al-Mamun M M, Islam MS, Ahmed MU, Kabir ER (2017). Association of BRCA1, BRCA2, RAD51, and HER2 gene polymorphisms with the breast cancer risk in the Bangladeshi population. Breast Cancer.

[64] Yang D, Khan S, Sun Y, Hess K, Shmulevich I, Sood AK (2011). Association of BRCA1 and BRCA2 mutations with survival, chemotherapy sensitivity, and gene mutator phenotype in patients with ovarian cancer. JAMA.

[65] Yang C, Zhang Z, Tang X, Zhang X, Chen Y, Hu T (2022). Pan-cancer analysis reveals homologous recombination deficiency score as a predictive marker for immunotherapy responders. Hum Cell.

[66] Xu X, Ma J, Yu G, Qiu Q, Zhang W, Cao F (2021). Effective predictor of colorectal cancer survival based on exclusive expression pattern among different immune cell infiltration. J Histochem Cytochem.

[67] Mehraj U, Ganai RA, Macha MA, Hamid A, Zargar MA, Bhat AA (2021). The tumor microenvironment as driver of stemness and therapeutic resistance in breast cancer: new challenges and therapeutic opportunities. Cell Oncol (Dordr).

[68] Waniczek D, Lorenc Z, Śnietura M, Wesecki M, Kopec A, Muc-Wierzgoń M (2017). Tumor-associated macrophages and regulatory T cells infiltration and the clinical outcome in colorectal cancer. Arch Immunol Ther Exp (Warsz).

[69] Faget J, Sisirak V, Blay J Y, Caux C, Bendriss-Vermare N, Ménétrier-Caux C (2013). ICOS is associated with poor prognosis in breast cancer as it promotes the amplification of immunosuppressive CD4(+) T cells by plasmacytoid dendritic cells. Oncoimmunology.

[70] Yin X, Wang Z, Wang J, Xu Y, Kong W, Zhang J (2021). Development of a novel gene signature to predict prognosis and response to PD-1 blockade in clear cell renal cell carcinoma. Oncoimmunology.

[71] Petersen RP, Campa MJ, Sperlazza J, Conlon D, Joshi MB, Harpole DH (2006). Tumor infiltrating Foxp3+ regulatory T-cells are associated with recurrence in pathologic stage I NSCLC patients. Cancer.

[72] Chao JL, Savage PA (2018). Unlocking the complexities of tumor-associated regulatory T cells. J Immunol.

[73] Song P, Li W, Guo L, Ying J, Gao S, He J (2022). Identification and validation of a novel signature based on NK cell marker genes to predict prognosis and immunotherapy response in lung adenocarcinoma by integrated analysis of single-cell and bulk RNA-sequencing. Front Immunol.

[74] Gu-Trantien C, Loi S, Garaud S, Equeter C, Libin M, de Wind A (2013). CD4^+^ follicular helper T cell infiltration predicts breast cancer survival. J Clin Invest.

[75] Timperi E, Pacella I, Schinzari V, Focaccetti C, Sacco L, Farelli F (2016). Regulatory T cells with multiple suppressive and potentially pro-tumor activities accumulate in human colorectal cancer. Oncoimmunology.

